# Comment on: Effects of *Nigella Sativa* on Type-2 Diabetes Mellitus: A Systematic Review

**DOI:** 10.3390/ijerph17051630

**Published:** 2020-03-03

**Authors:** Sherif T. S. Hassan, Miroslava Šudomová

**Affiliations:** 1 Department of Applied Ecology, Faculty of Environmental Sciences, Czech University of Life Sciences Prague, Kamýcká 129, 6-Suchdol, 165 21 Prague, Czech Republic; 2 Museum of Literature in Moravia, Klášter 1, 664 61 Rajhrad, Czech Republic; sudomova@post.cz

In 2015, the Nobel Prize in Physiology or Medicine was awarded to Youyou Tu, for her discovery of the natural anti-malarial drug Artemisinin. Since then, natural products research has earned great attention, in terms of the significance of their role as a vast source of new drugs in modern drug discovery research. Over the past few decades, systematic reviews and meta-analyses have become crucial in healthcare settings. Clinicians and healthcare providers use these types of research to keep up to date with their field. Moreover, these reviews are frequently adopted as a starting point for improving clinical practice guidelines. 

Therefore, and with a great interest in the latest advances in natural products research, with emphasis on the induced pharmacological properties, we recently read a systematic review that has been published in the *International Journal of Environmental Research and Public Health* by Hamdan et al. [1]. After reading the article, we would like to address several critical concerns and comments, which were ascertained to be essential for such a type of review article. Unfortunately, numerous methodological flaws were observed, which could have a marked impact on the quality and the value of the review and its outcomes, as well as the potential applicability in complementary medicine.

Although Hamdan et al. recovered the analyzed articles from two major online databases (Scopus and Medline), their search was restricted to the English language, cross-sectional study designs, and only two databases. These constraints limit the search procedure and increase the probability of missing numerous relevant studies. Thus, the comprehensiveness of the outcomes of this review could have been enhanced through assembling the data from articles reported in various languages [2], as well as employing other study designs. Additionally, various other online databases could be utilized to collect more relevant data [3]. In this systematic review, the authors non-systematically selected articles with broad and heterogeneous methodological designs, including randomized control trials and observational investigations with no obvious selection criteria regarding the methodological designs of the studies involved. Furthermore, the authors failed to evaluate the quality of the evidence, the risk of bias and the heterogeneity of the incorporated studies.

Most importantly, this review is not reported according to the Preferred Reporting Items for Systematic Reviews and Meta-Analysis (PRISMA) statement guidelines, where no proof was presented [4]. In addition, there was no confirmation to indicate that the used protocol (the search strategy, determination of eligibility, extraction, and analysis for the systematic review) was registered in any database of systematic review protocols [5]. Such procedures could reduce bias and improve the quality of the review by certifying the recorded outcomes and the level of evidence, as well as support future updates. To sum up, the quality of the evidence along with the accuracy of reported conclusions were significantly affected by the observed methodological flaws. Eventually, and based on the above-mentioned concerns, we highly recommend that the authors consider and follow the guidelines such as those recommended by Siddaway et al. [6] in their future work to produce a high-quality systematic review.
